# Ovarian down Regulation by GnRF Vaccination Decreases Reproductive Tract Tumour Size in Female White and Greater One-Horned Rhinoceroses

**DOI:** 10.1371/journal.pone.0157963

**Published:** 2016-07-12

**Authors:** Robert Hermes, Franz Schwarzenberger, Frank Göritz, Serena Oh, Teresa Fernandes, Rui Bernardino, Antoine Leclerc, Eva Greunz, Abraham Mathew, Sarah Forsyth, Joseph Saragusty, Thomas Bernd Hildebrandt

**Affiliations:** 1 Leibniz Institute for Zoo and Wildlife Research, PF 700430, D-10342 Berlin, Germany; 2 University of Veterinary Medicine, Biomedical Sciences, Veterinärplatz 1, 1210 Vienna, Austria; 3 Singapore Zoological Gardens, 80 Mandai Lakeroad, Singapore 729826, Singapore; 4 Lisbon Zoo, Veterinary Hospital, Estrada de Benfica, n°158-160 Lisbon, Portugal; 5 ZooParc de Beauval, 41110 Saint-Aignan, France; 6 Thoiry Zoo, 78770 Thoiry, France; 7 Colchester Zoo, Maldon Road, Stanway, Essex CO3 0SL, United Kingdom; Faculty of Animal Sciences and Food Engineering, University of São Paulo, BRAZIL

## Abstract

Reproductive tract tumours, specifically leiomyoma, are commonly found in female rhinoceroses. Similar to humans, tumour growth in rhinoceroses is thought to be sex hormone dependent. Tumours can form and expand from the onset of ovarian activity at puberty until the cessation of sex-steroid influences at senescence. Extensive tumour growth results in infertility. The aim of this study was to down regulate reproductive function of tumour-diseased and infertile females to stop further tumour growth using a Gonadotropin releasing factor (GnRF) vaccine. Four infertile southern white (*Ceratotherium simum simum*) and three Greater one-horned rhinoceroses (*rhinoceros unicornis*) with active ovaries and 2.7 ± 0.9 and 14.0 ± 1.5 reproductive tract tumours respectively were vaccinated against GnRF (Improvac^®^, Zoetis, Germany) at 0, 4 and 16 weeks and re-boostered every 6–8 months thereafter. After GnRF vaccination ovarian and luteal activity was suppressed in all treated females. Three months after vaccination the size of the ovaries, the number of follicles and the size of the largest follicle were significantly reduced (P<0.03). Reproductive tract tumours decreased significantly in diameter (Greater-one horned rhino: P<0.0001; white rhino: P<0.01), presumably as a result of reduced sex-steroid influence. The calculated tumour volumes were reduced by 50.8 ± 10.9% in Greater one-horned and 48.6 ± 12.9% in white rhinoceroses. In conclusion, GnRF vaccine effectively down regulated reproductive function and decreased the size of reproductive tract tumours in female rhinoceros. Our work is the first to use down regulation of reproductive function as a symptomatic treatment against benign reproductive tumour disease in a wildlife species. Nonetheless, full reversibility and rhinoceros fertility following GnRF vaccination warrants further evaluation.

## Introduction

In captive rhinoceros species, reproductive tract tumours such as leiomyoma, adenoma or adenocarcinoma have been frequently reported [1,2,3,4,5,6]. Whereas single reports on uterine adenoma and adenocarcinoma suggest low incidence of epithelial cell tumours [5], a large number of reports on reproductive tract leiomyomas across multiple rhinoceros species suggests an increased incidence of these benign, smooth muscle cell tumours. In one study group of twenty-five Greater one-horned rhinoceros the incidence of reproductive tract leiomyoma in females older than 12 years of age was an astonishing 100% [3]. Consequently, leiomyomas are regarded as a major contributing factor for reduced female fecundity in Greater one-horned rhinoceros. Reproductive tract tumours discovered in Sumatran rhinoceros (ssp.) captured from the wild demonstrated that these reproductive tract tumours also occur in wild rhinoceros populations [Hildebrandt et al., unpublished data].

While leiomyoma are small in size and number to begin with, they grow and increase in numbers over time. After decades of hidden tumour growth, bloody vaginal discharge or tumours extruding from the vagina are the end stage clinical symptoms of this largely asymptomatic disease in rhinoceroses [3]. When diagnostic ultrasonography identifies reproductive tract tumours, the underlying tumour aetiology may explain previous fertility issues. Single large tumours, tumour necrosis, complete vaginal and cervical obstruction or compression of ureter, urethra or rectum by tumour masses may also explain observed pain reactions during mating, conception failure, recurrent miscarriage, stillbirth, bloody discharge, anaemia, or problems associated with urination or defecation [3]. All of the above complications are also commonly observed in humans [7,8,9] where the cumulative prevalence of intramural leiomyoma by the time of menopause is 70–80% [7,10,11,12].

Historically, it has been difficult to manage female rhinoceros that suffer from reproductive tract tumours sometimes from as early as 13 years of age [3]. In females with only few and small tumours the promotion of breeding to prevent further tumour growth seems the best and most pragmatic choice. This recommendation is in analogy to humans, where growth of leiomyomas is restricted during pregnancy [12]. Furthermore, pregnancy appears to exert a certain protective effect, reducing the risk of developing new leiomyoma. Multiparity further reduces the risk of developing leiomyomas by 20 to 40% per parity [11,13,14]. Thus, humans becoming pregnant early in life and delivering multiple offspring have the lowest probability of developing leiomyoma or, if such have developed, their growth rate is considerably reduced. Similar to humans, recent data from Greater one-horned rhinoceros suggest that females starting to breed at a young age show a twofold higher fecundity compared to females starting to breed later in life. This higher fecundity in early breeders has been explained by the protective effect of pregnancy, lactation and multiparity preventing reproductive tumour development and growth [3].

But what are the options for females when a single large or an excessive number of tumours is found? If exempted from reproduction, these females often develop complications secondary to progressive tumour growth. Surgical interventions such as tumour removal, tumour embolism or complete ovarhysterectomy, commonly applied in domestic species and humans, are challenging in rhinoceroses and are not currently viable treatment options. The rhinoceros’ extended ribcage, tough skin, fibroelastic peritoneal lining, and limited post-surgical care complicate extensive standard laparoscopic or surgical interventions. One report of an ovariohysterectomy attempted under general anaesthesia on one Greater one-horned rhinoceros proved fatal because of such difficulties [6]. If surgical tumour resection is excluded, the ideal aim of a therapy would be to prevent further tumour growth and development, thus improving long-term animal welfare.

In domestic species and humans, the use of a vaccine against gonadotropin releasing factor (GnRF) to down regulate reproductive function has been studied extensively. The GnRF analogue in the vaccine is bound to a carrier protein of a corynebacterium. Antibodies produced in response to the vaccination eliminate the intrinsic GnRF. In absence of GnRF, FSH and LH hormone release from the pituitary gland is suspended and thus reproductive function in males and females is down regulated. In males, the GnRF analogue vaccine is commercially used in boars to reduce boar taint as an alternative to surgical castration [15,16]. In men, it is used in advanced prostate cancer patients to influence hormone dependent tumour growth [17]. In females, the immune induced suspension of FSH and LH release from the pituitary down regulates ovarian function. In the mare, immunization against GnRF suppresses ovulation, ovarian activity and oestrous behaviour [18,19]. The suppression of reproductive function by GnRF vaccination shows effect eight weeks after initial immunization but remains reversible in up to 92–100% of treated mares [18,20].

Specifically due to the reversibility of its effects and biosafety on existing pregnancies, GnRF vaccine is promising as a contraceptive for managing overabundant wildlife populations and has been tested in various artiodactyl species such as the elk, the bison or the white tailed deer [21,22,23]. In elephants, the largest mammal in which the seemingly universal GnRF vaccine has been tested, results were non-uniform. While in male elephants of both Asian and African species and in female Asian elephants the immunization induced down regulation of reproductive function, it has so far failed to induce anoestrous in free ranging female African elephants [24,25,26,27]. Because of a lack of specific tumour treatments or practical surgical options in rhinoceroses, our goal was to investigate the use of GnRF vaccine in female rhinoceros with extensive reproductive tract tumours. In the absence of viable treatment options, GnRF vaccination was aimed at improving the animal’s welfare by down regulating ovarian function and suppressing hormone-dependent reproductive tumour growth.

## Methods and Materials

### Ethics statement

This study was conducted on captive Indian and white rhinoceroses in four zoos, species that are listed by the IUCN as vulnerable and near threatened, respectively. The study was carried out in strict accordance with the German National Protection of Animals Act from 24.07.1972 and its last revision from 15th July 2009. Under this Act, an examination directed toward diagnosing and treating an animal’s disease is not defined as an animal experiment (§7) but as a mandatory act of animal welfare. Under these circumstances no specific animal welfare or ethics permissions were required. Animals were examined either voluntarily or under sedation for the otherwise painless transrectal ultrasound examinations. All animal holders were in agreement of the study. Further under this Act (§5) anaesthesia is mandatory only if comparable procedures in humans require anaesthesia. Gynaecological examinations or vaccinations as performed in this study do not require anaesthesia in humans, and technically do not require anaesthesia in rhinoceros either, if animals are properly conditioned. However, captive wild animals do not comply always with veterinary diagnostic procedures. To achieve a level of tolerance to the non-harmful, painless gynaecological ultrasound examination, sedation or anaesthesia was performed in most females in this study. No further specific permissions were required for diagnostic ultrasound or vaccination of these captive animals at any of the institutions involved. Although not mandated by law, the study design was approved by IACUC animal ethics committee of the Leibniz Institute for Zoo and Wildlife Research (Permit number: 2009-04-19).

### Animals and treatment

Seven captive, female rhinoceroses (n = 4, *Ceratotherium simum simum*; n = 3, *Rhinoceros unicornis*) 24–44 years of age, previously diagnosed with extensive reproductive tract tumours by ultrasonography and exempted from reproduction were selected for this study (Table 1). All females had ovarian and irregular luteal activity before the GnRF immunisation was applied, yet only two ever had offspring. In four animals, frequent haemorrhagic vaginal discharge had been reported.

**Table 1:** Female white (*ceratotherium simum simum*) and Greater one-horned (GOH) (*rhinoceros unicornis*) rhinoceroses treated with GnRF vaccine.

Animal	Species	Studbook number	Birth year	Age (y)	Oestrous cycle	Offspring	Diagnosis Reproductive tract	Luteal activity cessation (mo)	duration of cessation (mo)
1	white rhino	607	1970	39	yes	no	Tumors, discharge	2,0	34
2	white rhino	653	1981	32	yes	no	Tumors, discharge	3,5	10
3	white rhino	138	1968	44	yes	no	Tumors, discharge	2,5	13
4	white rhino	291	1970	42	yes	no	Tumors	3,0	18
5	Indian rhino	93	1979	32	yes	yes	Tumors	2,0	12
6	Indian rhino	161	1989	24	yes	yes	Tumors, discharge	n.d.	8
7	Indian rhino	97	1979	34	yes	no	Tumors	n.d.	8

n.d.: no determined due to phytoprogestins / phytochemicals cross reacting with hormone essay.

GnRF vaccination using Improvac^®^, a synthetic GnRF-peptid-analogue, has been effective in boar at a dose of 300μg and in mares at a dose of 400μg [15,16,18,19,20]. Yet, in much larger mammals, such as elephants, GnRF immunisation has been effective only after administration of higher doses than those used in the domestic species [25,26,27]. Therefore the dose of GnRF vaccine used here in rhinoceroses followed efficient dose recommendations reported from elephants. Females were vaccinated with 450μg of gonadotropin releasing factor (GnRF) analogue at 0, 4 and 16 weeks for initial immunisation by either hand injection, pole syringe or dart. Further boosters were given in 6–8 month intervals. The vaccine (Improvac^®^, Zoetis Deutschland GmbH, Berlin, Germany) contained 150 μg/mL synthetic GnRF-peptid-analogue, conjugated with diphtheria toxoid. The adjuvants, was composed of 150mg/ml diethylaminoethyl (DEAE)-Dextran in aqueous solution.

### Endocrinology

The effect of the GnRF vaccination was monitored by faecal steroid analysis over a minimum period of 12 months and up to 48 months. Faecal samples were collected one to two times per week throughout treatment and stored at -20°C until analysed. Faecal steroid metabolite concentrations were analysed using group-specific enzyme-immunoassays as described previously [28,29,30,31]. Previous publications report that such types of assays can be used for faecal steroid metabolite analysis in the majority of mammalian species, provided that species specific differences in steroid hormone metabolism are taken into account [28]. Therefore in samples from white rhinoceroses 20-oxo-pregnane concentrations were analysed, whereas samples from the Greater one-horned rhinoceroses were analysed for 20α-OH-pregnanes and for oestrogen precursor concentrations. 20α-OH-pregnanes were analysed using a pregnanediol assay, whereas oestrogen precursors were analysed using a 17-oxo-androstane assay [29]. The assays were validated by confirming parallelism between standard curves and serial dilutions of the faecal extracts. The intra- and inter-assay coefficients for the assays were between 10 and 15%. Hormone concentrations are presented as ng/g wet faeces. In addition to faecal steroid analysis, 20-oxo-pregnanes were analysed in selected serum samples in two Greater-one horned rhinoceroses. The plasma samples were analysed using the same 20-oxo-pregnane assay as was used for analysis of faecal samples from the white rhinoceros. In the 20-oxo-pregnane assay progesterone is used as the standard substance. This assay was previously established for monitoring luteal function in blood samples of non-pregnant and pregnant white rhinoceros [32]. Despite ultrasonographic detection of suppression of ovarian activity, high baseline fecal 20α-OH-pregnane concentrations were observed in two female Greater-one horned rhinoceroses from one of the institutions. Therefore the influence of nutritional components on the endocrine results was tested. One set of dried feed samples (assorted local leaves, herbivore pellets (J.T. Johnson & Sons, Kapunda, South Australia), meadow hay (Tallahesse pte Ltd, Singapore), Napier grass (pennisetum purpureum), and wheat (triticum aestivum) was tested for their cross reactivity with the group-specific 20α-OH-pregnane assay. For this, dried feed samples were extracted and analyzed as if they were regular faecal samples. The parallelism between the assay standard curve and serial dilutions of the fecal extracts was evaluated.

### Ultrasonography

To evaluate the effect of GnRF vaccination on the reproductive tract organs and tumours, the vagina, cervix, uterus and ovaries of six females were examined by standardized ultrasonography [3,4,30]. One white rhinoceros was not available for ultrasonographic evaluations. For comparison of effects before and after treatment, genital organs and reproductive tumours were imaged (VOLUSON I, 2–5 MHz, GE Healthcare, Berlin, Germany). To evaluate ovarian activity before and after vaccination in female rhinoceros several ovarian parameters were measured. For this, the maximum ovarian diameter, total number of follicles, the diameter of the largest follicle and the presences of corpora lutea were measured in each female at a random point of the ovarian cycle before and >3 months after treatment. The parameters were chosen in analogy to a previous GnRF vaccination study in mares [18]. In addition, dimensions of individual and well distinguished tumour masses were measured before and >3 months after start of the vaccination regime. To determine the growth rate of the reproductive tract tumours, tumour growth was assessed over a period of two months in one Greater one-horned rhinoceros before immunocontraception treatment started. For reproductive tract ultrasonography, three animals were trained to tolerate the procedure without chemical restraint. In all other animals standing sedation was required [33,34]. The choice of sedation or of voluntary examination was adjusted to the individual rhinoceros. This choice, however, had no impact on the data collected.

### Statistical analysis

Statistical analysis was performed using GraphPad InStat software (GraphPad Software Inc, Version 3.00, San Diego, CA, USA). Reproductive tumour data was compared pairwise before and after vaccination for each individual. To compare means, a Wilcoxon two-tailed matched pairs test was performed. All values are reported as mean ± SEM. Differences were considered significant when *P* < 0.05. Endocrine results from the four white rhinoceroses studied were grouped in monthly intervals starting with the day of vaccination. Because results were not normally distributed, median values have been created and depicted in a boxplot graph. The number of samples in each box ranged between 9–17 samples.

## Results

### Ultrasonography prior vaccination

**Fig 1:**
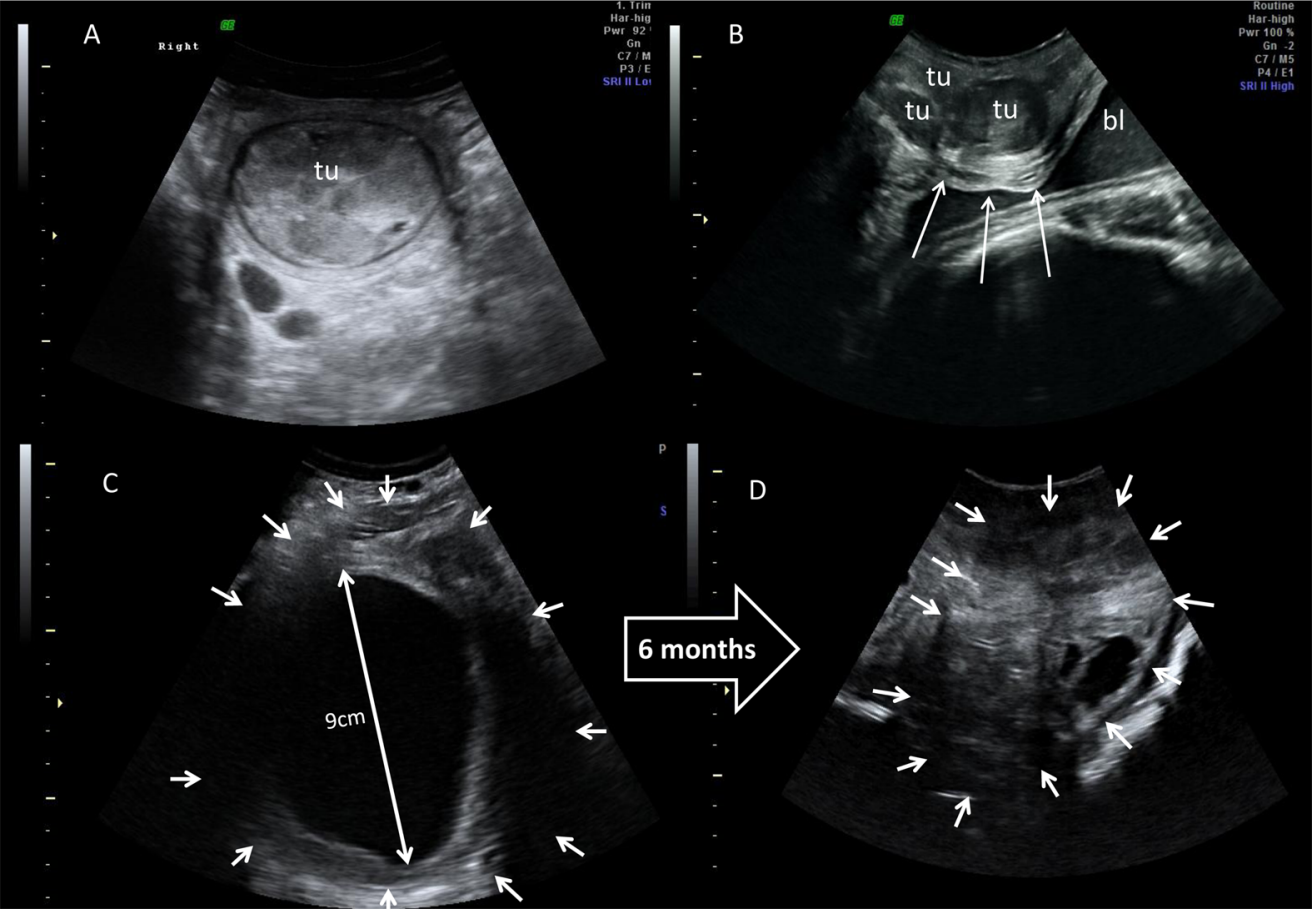
Ultrasonographic images. A: Solid uterine tumour (tu) in a white rhinoceros. B: multiple vaginal tumours (tu) in a Greater one-horned rhinoceros. The largest tumour compresses the ureter (↓) above the bladder (bl). C + D: Ovary of a Greater one-horned rhinoceros before and 6 months after GnRF vaccination. Dimensions of the ovary (←) and follicular activity regressed notably after the vaccination. Note large physiological diameter of the dominant follicle and small and irregular shaped atretic follicles before and after vaccination. Standard scales of 1 cm are imprinted on the side of the ultrasound images.

**Fig 2:**
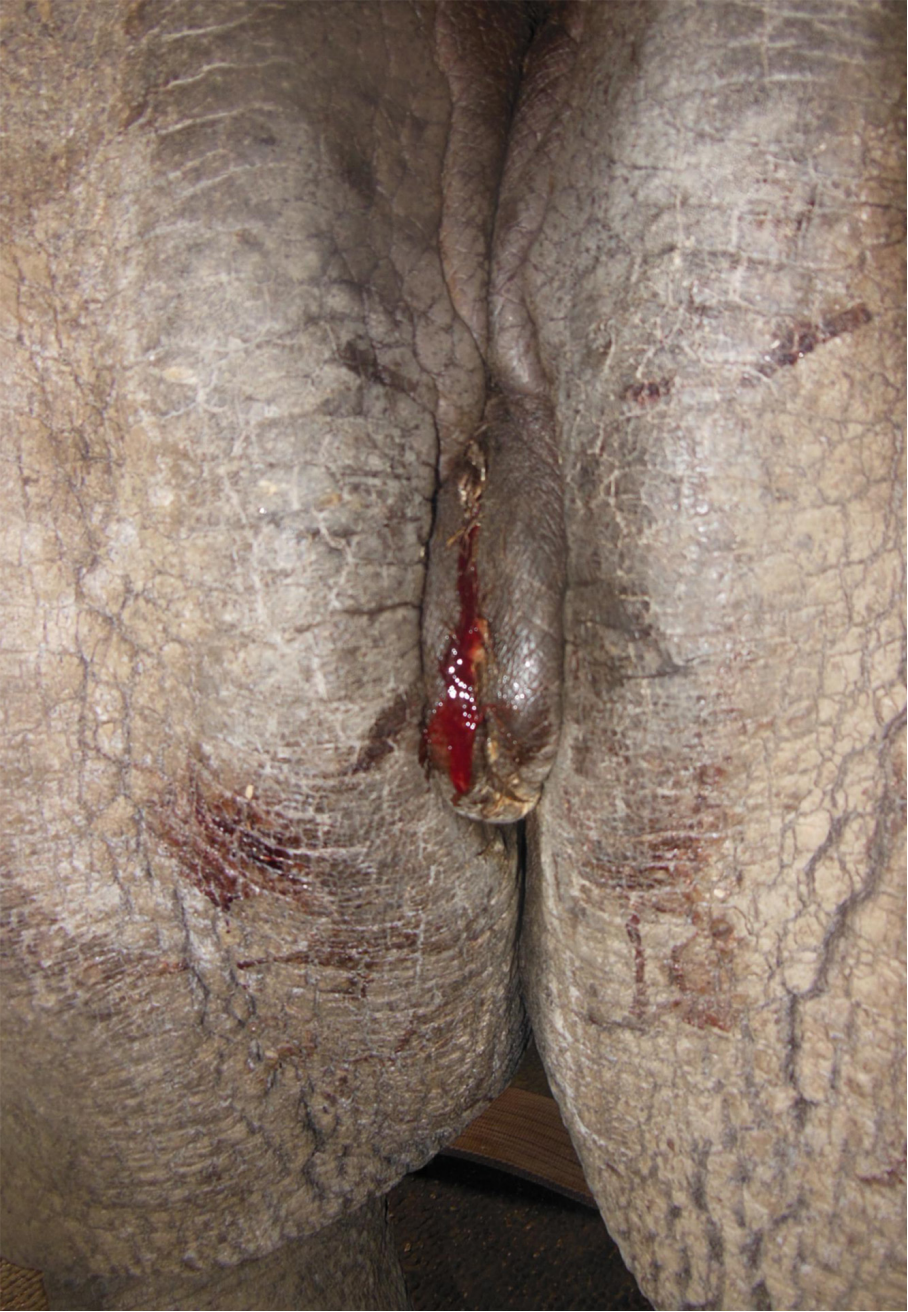
Bloody vaginal discharge in a white rhinoceros with uterine leiomyoma. Erratic vaginal discharge is one of the few clinical symptoms indicating the presence of advanced reproductive tumours.

Ovaries in all females examined were active prior to GnRF vaccination. Ovarian size, follicles and /or corpus luteum (CL) indicated the active ovarian status as confirmed by luteal activity in the faecal hormone analysis (Fig 1, Table 2). Prior to vaccination, Greater one-horned and white rhinoceroses carried a mean number of 14.0 ± 1.5 and 2.7 ± 0.9 reproductive tract tumours, respectively. The mean size of these tumours in Greater one-horned and white rhinoceroses was 3.6 ± 0.3 cm and 4.7 ± 0.8 cm, respectively. The incidence of reproductive tract tumours was higher in Greater one-horned compared to white rhinoceroses (Table 3). In the Greater one-horned rhinoceroses, tumours were identified throughout the genital tract in the vagina, cervix and uterus, while in white rhinoceroses tumours were situated in the uterus only (Fig 1). Tumours were less echogenic in relation to the reproductive organs they were located in. Few larger tumours had a high echogenic centre, indicating tumour tissue necrosis. In the Greater one-horned rhinoceros, vaginal and cervical tumours had merged, forming a conglomerate of tumour masses. Vaginal tumours in two Greater one-horned rhinoceros females, one of which was reported to have become overly aggressive, were in close proximity to the ureter (Fig 1), causing ureter compression and retrograde ureter dilation. Bloody, vaginal discharge, the only clinical symptom of this tumour disease, was observed in four females (Fig 2). Necrotic tumour masses in these animals were interspersed and surrounded with fluid filled cavities.

**Table 2:** Ovarian activity in white and Greater one-horned (GOH) rhinoceroses before and after GnRF vaccination.

Species / Parameter	Before GnRF vaccine	After GnRF vaccine	Significance (*P*)
**white rhinoceros (n = 3)**			
Ovarian diameter (cm)	6,5 ± 0,2	4,6 ± 0,3	< 0,003
Follicles (n)	3,6 ± 1,1	0,3 ± 0,2	< 0,03
Largest follicle (cm)	0,9 ± 0,1	0,2 ± 0,1	< 0,03
Number of CL (n)	0,3 ± 0,2	0.0 ± 0,0	n.c.
**GOH rhinoceros (n = 3)**			
Ovarian diameter (cm)	12,0 ± 0,6	7,3 ± 0,6	< 0,03
Follicles (n)	1,8 ± 0,5	0,5 ± 0,2	< 0,03
Largest follicle (cm)	3,9 ± 1,5	0,5 ± 0,3	< 0,03
Number of CL	0,7 ± 0,2	0.0 ± 0,0	n.c.

Statistical test used: Wilcoxon matched pairs test. *P* level of significance *P* < 0.05. n.c.: not calculated as one value is zero

**Table 3:** Change of reproductive tract tumours in white and Greater one-horned rhinoceroses (GOH) under GnRF vaccine measured by ultrasonography.

animal	species	tumours	mean tumour diameter			mean tumour volume			
		(#)	prior GnRF vaccine (cm)	after GnRF vaccine (cm)	decrease (cm)	before GnRF vaccine (cm^3^)	after GnRF vaccine (cm^3^)	decrease (cm^3^)	decrease (%)
1	white rhino	1	5,5	3,5	2,0	87,1	22,4	64,7	74,2
2	white rhino	4	5.5 ± 0.6	4,7	0.8 ± 0.3	95,1 ± 23,3	60,5 ± 17,9	34,6 ± 16,6	33,8 ± 10,5
4	white rhino	3	3.0 ± 0.6	2,6	0.4 ± 0.1	19,0 ± 8,7	12,8 ± 5,3	6,1 ± 3,5	37,9 ± 10,6
**Mean**		**2,7 ± 0,9**	**4,7 ± 0,8**	**3,6 ± 0,6**	**1,1 ± 0,5**	**67,1 ± 24,2**	**31,9,7 ± 14,6**	**35,1 ± 16,9**	**48,6 ± 12,9**
5	GOH rhino	12	3.9 ± 0.4	3,1	0.9 ± 0.2	46,5 ± 14,6	20,9 ± 6,3	25,5 ± 8,7	46,9 ± 7,7
6	GOH rhino	17	3.0 ± 0.4	1,9	1.0 ± 0.2	28,6 ± 12,8	8,0 ± 3,6	20,7 ± 9,5	63,7 ± 6,1
7	GOH rhino	13	4.0 ± 0.8	3,4	0.7 ± 0.2	93,0 ± 51,0	65,1 ± 42,9	28,0 ± 13,0	41,9 ± 6,7
**Mean**		**14,0 ± 1,5**	**3,6 ± 0,3**	**2,8 ± 0,5**	**0,9 ± 0,2**	**56,0 ± 32,3**	**31,3 ± 28,7**	**24,7 ± 3,7**	**50,8 ± 10,9**
**Mean**	**combined**	**8,3 ± 2,7**	**4,2 ± 0,5**	**3,2 ± 0,4**	**1,0 ± 0,2**	**61,6 ± 14,0**	**31,6 ± 10,1**	**29,9 ± 8,0**	**49,7 ± 6,5**

Reproductive tract tumour size (n = 17) evaluated in one Greater one-horned rhinoceros over two months prior GnRF vaccination increased significantly from 2.5 ± 0.4 cm to 3.0 ± 0.3 cm (*P* = 0.0001) representing a tumour growth rate of 0.5 ± 0.1 cm within this period. Assuming these reproductive tumours had close to spherical structures their calculated mean volume increased from 19.6 ± 9.3 cm^3^ to 28.6 ± 12.8 cm^3^ equivalent to a mean tumour volume increase of 45.9 ± 18.9%.

### Ultrasonography after vaccination

The size of the ovaries, the number of follicles and the size of the largest follicle were significantly smaller (*P* < 0.002–0.03, Table 2, Fig 1C and 1D) after GnRF vaccination (n = 6). CLs as a marker of the ovarian cycle were not detected in any female after vaccination. The mean number of follicles decreased to ≤ 0.5 in in both species. The maximum size of single, remaining follicles on the ovaries after vaccination was ≤ 0.5 cm, 8.8% and 5% of the size of ovulatory follicles in white and Greater one-horned rhinoceroses, respectively (Table 2). Larger dominant follicles, which would be > 1.5 cm in white and > 5 cm in Greater one-horned rhinoceroses were completely absent after vaccination.

Under GnRF vaccination, reproductive tract tumours had decreased significantly in diameter by 1.1 ± 0.5 cm, from 4.7 ± 0.8 cm to 3.6 ± 0.6 cm, in white (*P* < 0.004) and by 0.9 ± 0.2 cm, from 3.6 ± 0.3 to 2.8 ± 0.5 cm, in Greater one-horned rhinoceroses (*P* < 0.0001) (Table 3). Ovarian down regulation, and thus the decreased sex-steroid hormone influence, reduced the calculated tumour volume by 48.6 ± 12.9% in white and by 50.8 ± 10.9% in Greater one-horned rhinoceroses (Table 3). This decrease in tumour volume was significant in both species but most significant in the Greater one-horned rhinoceroses (white: *P* < 0.004; Greater one-horned: *P* < 0.0001). Along with the decrease of tumour volume no additional new tumours have been observed in females examined 10–16 months after initial vaccination. Clinically, bloody vaginal discharge had ceased after the vaccination corresponding with the absence of fluids in or around the reproductive tract tumours.

### Faecal hormone analysis

**Fig 3:**
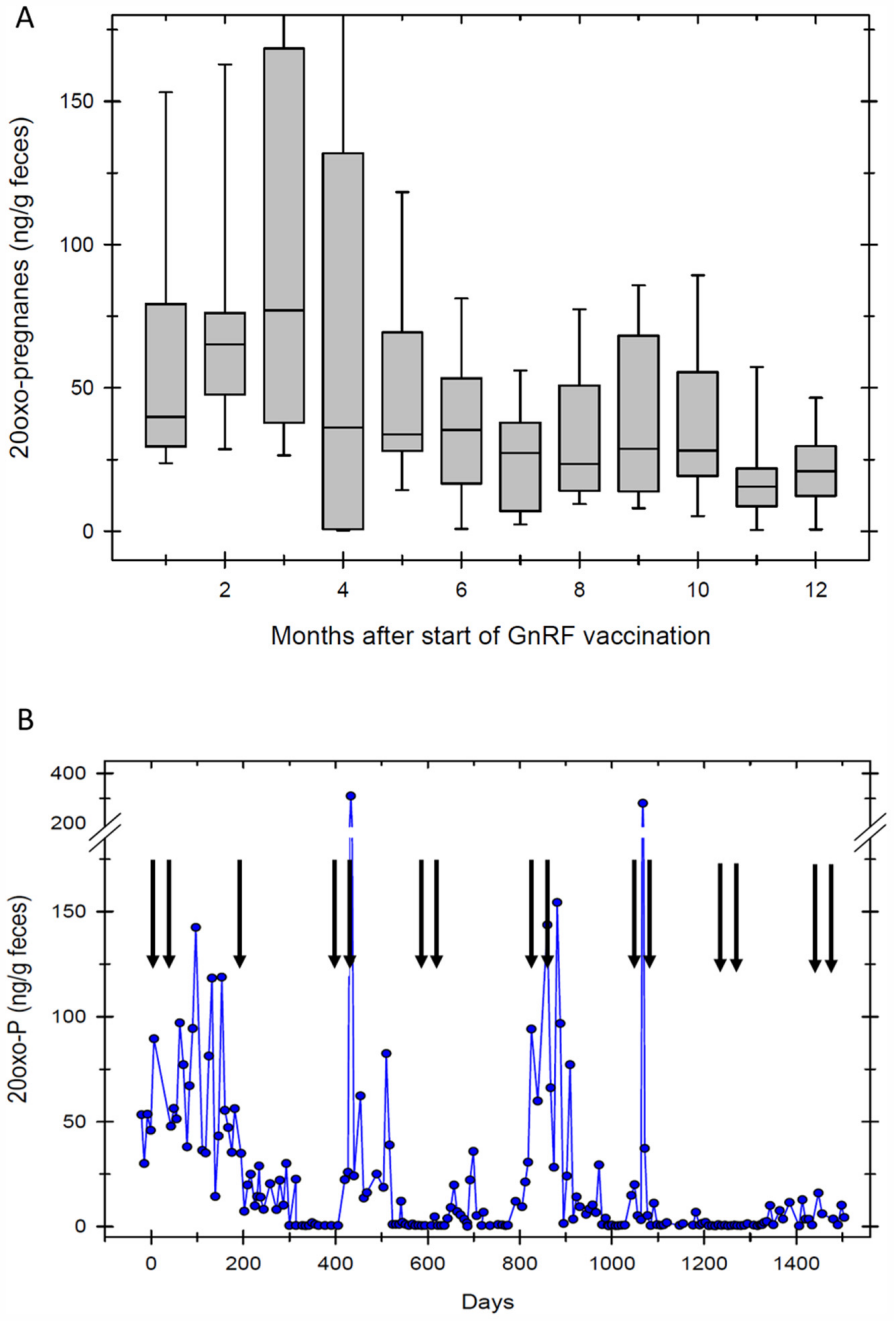
A: 20-oxo-pregnane concentrations in four white rhinoceroses vaccinated against GnRF. Endocrine results were grouped in monthly intervals and depicted in a boxplot graph. Luteal activity ceases after the initial vaccination. B. 20-oxo-pregnane concentrations in one white rhinoceros repeatedly boostered with GnRF vaccine (↓). Luteal activity ceases and resumes >400 days after the initial vaccination.

**Fig 4:**
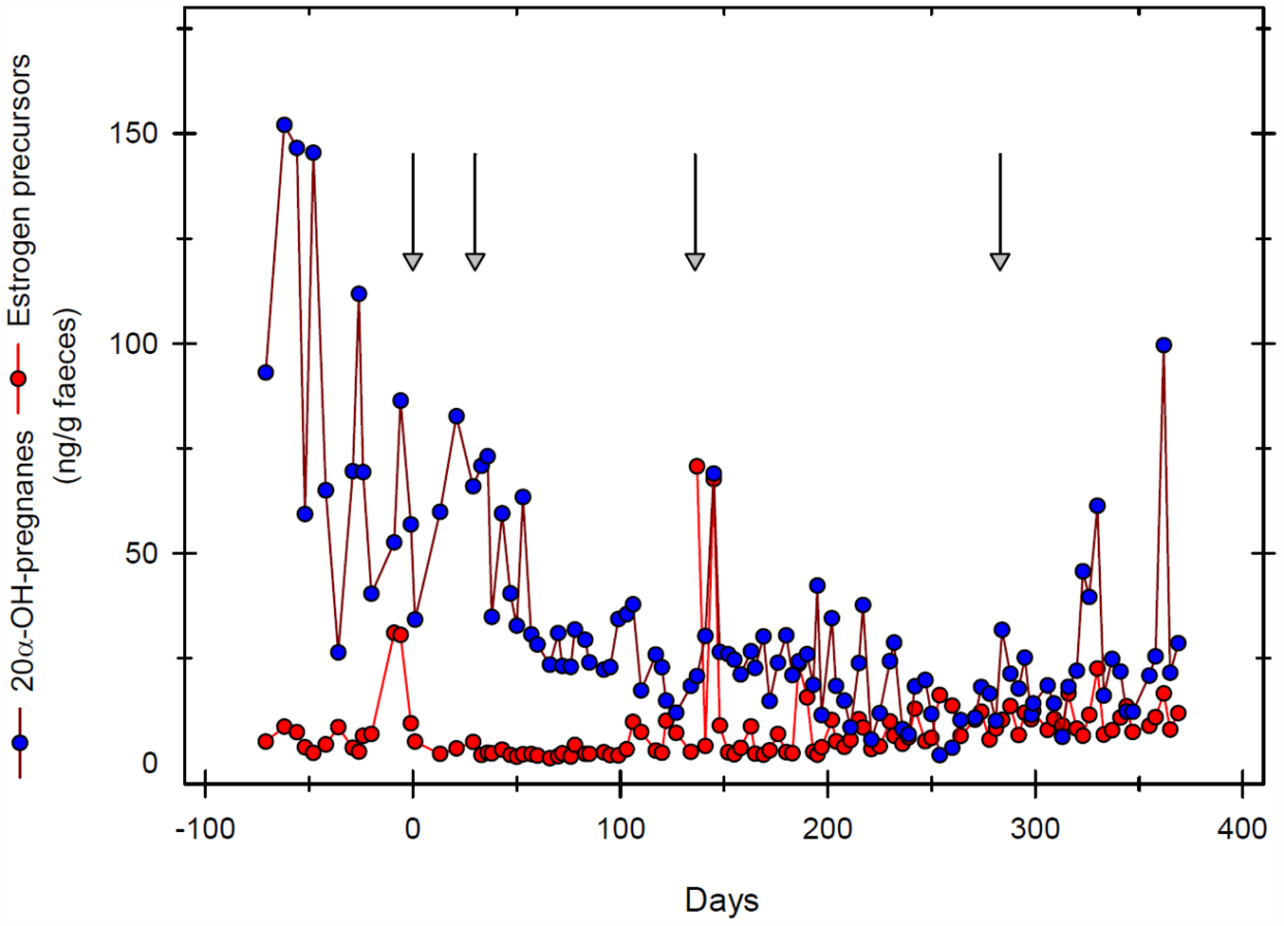
20α-OH pregnane and oestrogen precursor concentrations in one Greater one-horned rhinoceros before and after GnRF vaccination (↓). Luteal activity ceases after the initial vaccination. Low pregnane and oestrogen precursor concentrations rose shortly before and returned to baseline after the vaccine booster.

**Fig 5:**
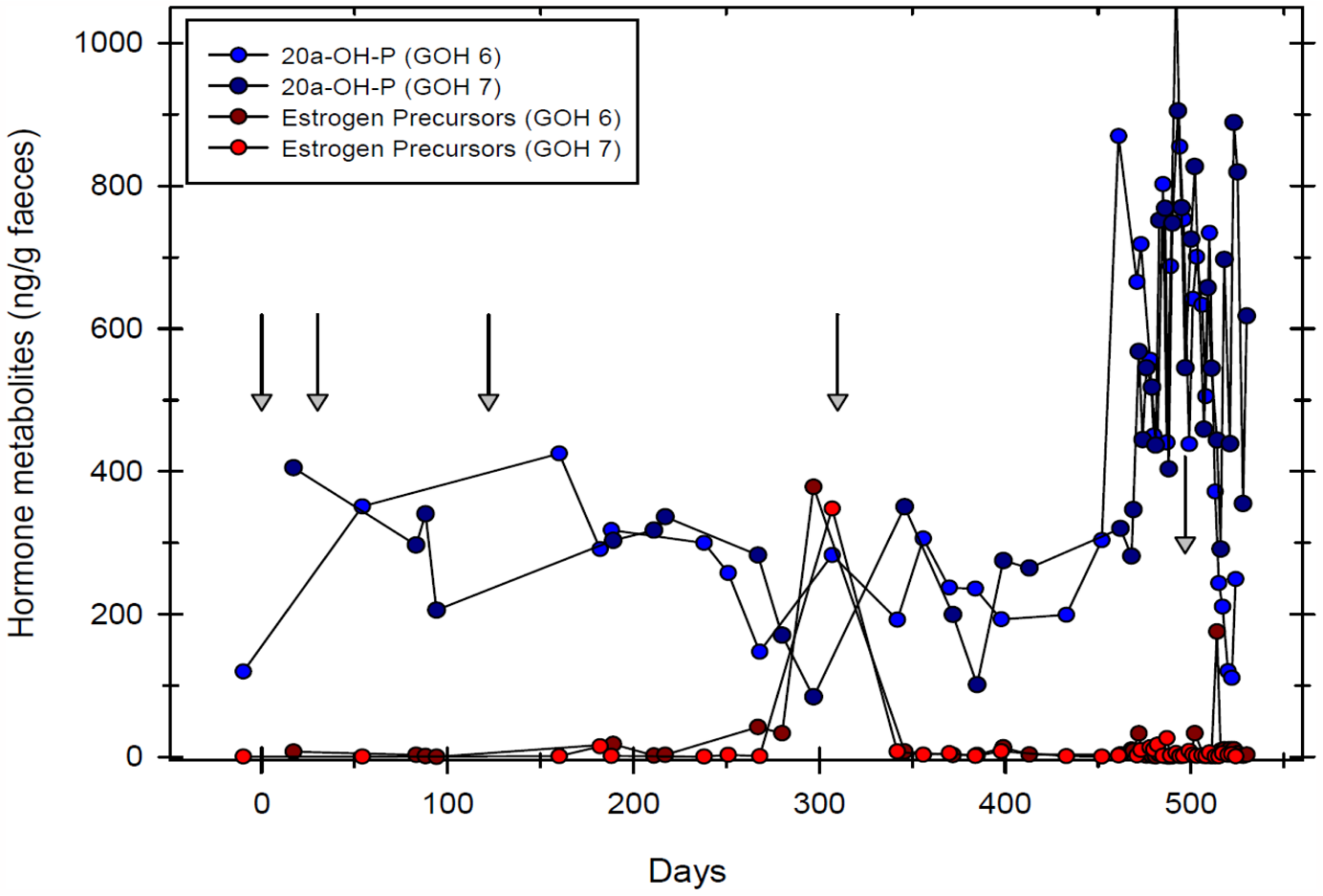
20α-OH-pregnane concentrations in two Greater-one horned rhinoceros repeatedly boostered with GnRF vaccine (↓). 20α-OH-pregnane concentrations in these two individuals was consistently > 100 ng/g or even > 400 ng/g faeces and did not correlate with ultrasonographically observed down regulation of ovarian activity.

In white rhinoceroses (n = 4) mean faecal 20-oxo-pregnane concentration, indicating ovarian luteal activity, decreased 4 months after initial GnRF vaccination (Fig 3). Luteal activity resumed after 6–8 months prior to vaccine booster in three out of four animals. In those females luteal activity had resumed, it decreased again 4 months after re-booster. In Greater one-horned rhinoceros, luteal activity measured by faecal 20α-OH-pregnane concentrations, also decreased 2 months after initial GnRF vaccination, corresponding with results from the white rhinoceros (Fig 4). However, baseline faecal 20α-OH-pregnane concentrations were measured in only one out of three vaccinated Greater one-horned rhinoceroses. In two Greater-one horned rhinoceroses consistently elevated, erratic concentrations of 20α-OH-pregnane did not match with ultrasonographically observed down regulation of ovarian activity (Fig 5). 20α-OH-pregnane concentrations measured in these two Greater one-horned rhinoceroses were consistently > 100 ng/g or even > 400 ng/g faeces, values normally measured only during pregnancy. In contradiction to high, faecal 20α-OH-pregnane concentration of ≥ 400 ng/g, values measured in a few selected serum samples were ≤ 1 ng/mL, indicating the cessation of luteal activity. To further evaluate ovarian activity in Greater one-horned rhinoceros precursor oestrogens were measured. Precursor oestrogens, an indicator of follicular activity prior ovulation, remained baseline in all Greater one-horned rhinoceroses for a period of 8 months after vaccination (Fig 5). An increase of oestrogen precursor concentrations starting 8 months after the first, initial vaccination indicated resumed ovarian activity. Re-booster vaccination thereafter induced cessation of precursor oestrogen activity again.

**Fig 6:**
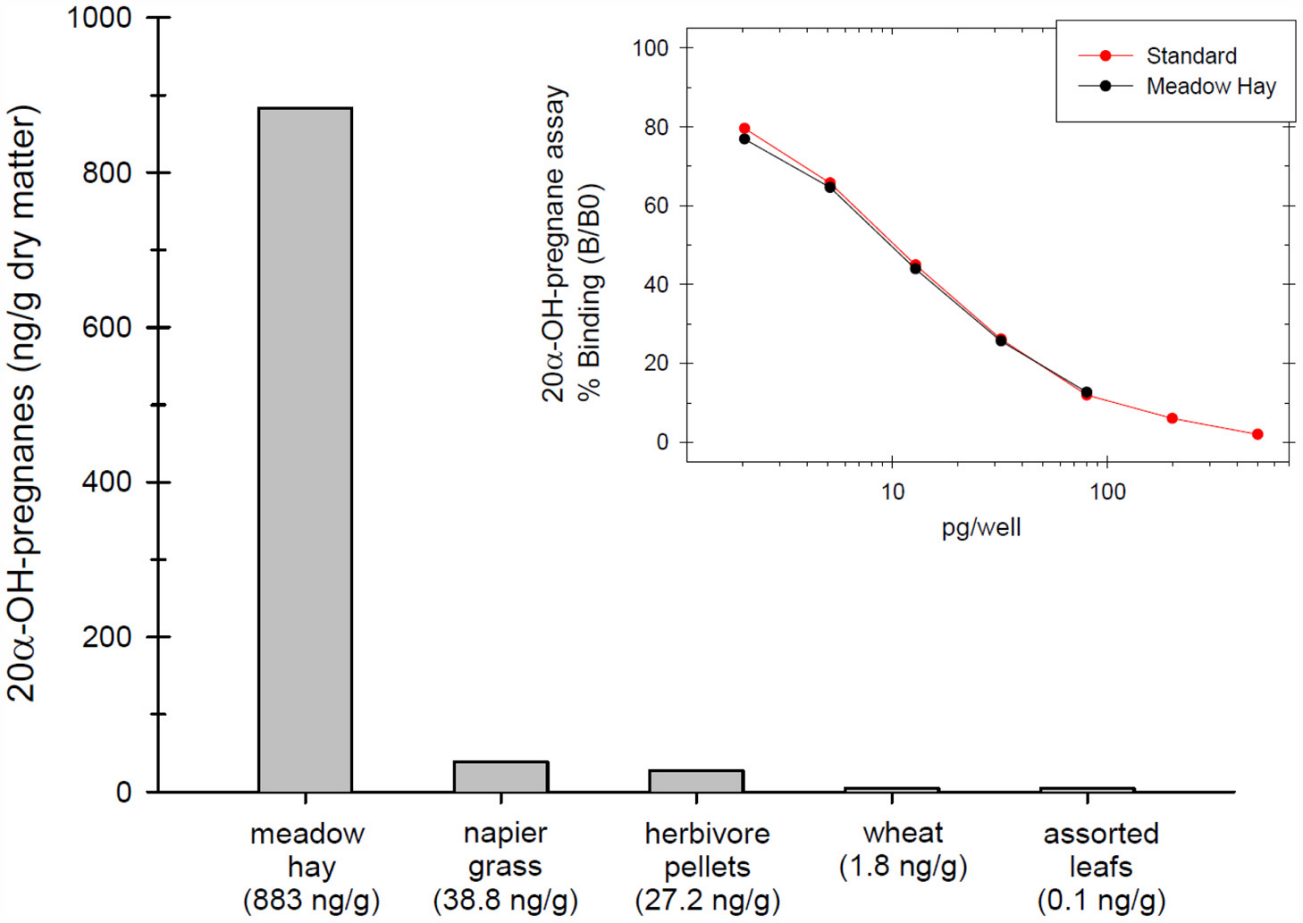
Because of the discrepancy of ultrasonographic and endocrine results of the two individual Greater-one horned rhinoceros of Fig 4, feed items given to these individuals were tested for cross reactivity cross reactivity with the group-specific 20α-OH-pregnane immunoassay. The inset figure shows dilutions of extracts of meadow hay in comparison to the 20α-OH-progesterone standard curve used in the immunoassay.

As faecal 20α-OH-pregnanes in two Greater-one horned rhinoceroses did not seem to match results obtained from serum 20-oxo-pregnanes, faecal pre-cursor oestrogens, and ultrasonography, we tested the possible influence of cross reacting substances in the feed on the group-specific enzyme immunoassay. Analysis of dried feed showed high cross reactivity of the group-specific 20α-OH-pregnane immunoassay with meadow hay and to much less extent with Napier grass and herbivore pellets in this institution (Fig 6). Dilutions of extracts of meadow hay ran in parallel to the 20α-OH-progesterone standard curve used in the immunoassay.

## Discussion

GnRF vaccine has been shown to down regulate reproductive function in various domestic and wildlife species of both sexes. In mares, female elk, bison, white tailed deer, and Asian elephant, immunization against GnRF was used as a contraceptive and successfully suppressed ovarian activity [21,22,23,25]. In this study, GnRF vaccine caused the suppression of ovarian and luteal activity in female white and Greater one-horned rhinoceroses. Ultrasonography documented a significant decrease in ovarian size, follicle number, size of the largest follicle and noted the absence of CLs in these rhinoceroses comparable to results from GnRF vaccinated mares [18]. Presumably, the GnRF analogue vaccine induced sufficient immune response against intrinsic GnRF to suspend FSH and LH release from the pituitary gland and to suppress ovarian function for a period of at least 5 months in female white and Greater-one horned rhinoceroses. In three females, ovarian activity resumed after 6–8 months suggesting a difference in their individual immune response to the vaccine. Restoration of luteal activity indicated the reversibility of the down regulatory effect of the GnRF analogue vaccine in female rhinoceros. If the aim of GnRF vaccination is not to resume luteal activity again, a shorter booster interval of about 5 months would be preferred over the 6–8 month time period used in this study.

In 1966, Charles Huggins was awarded the Nobel prize for his work on endocrine-induced regression of cancer tumours [35]. Huggins was the first to show the beneficial influence of hormonal down regulation on sex hormone-dependent reproductive tract tumours. Today, almost 60 years later, vaccination of men against GnRF has become a treatment option for hormone dependent prostate cancer [17]. In women, uterine leiomyoma is another example of a hormone dependent tumour. Leiomyoma and related symptoms have been treated by long term hormonal down regulation using long-term GnRH agonist formulations [36]. A six month treatment with a GnRH agonist in women decreased uterine volume by 45% and resolved leiomyoma related symptoms, specifically menorrhagia [36]. Our intention in infertile rhinoceroses with extensive reproductive tract tumours was similar. Down regulation of ovarian activity, and thus cessation of sex-steroid production targeted the presumably hormone-dependent tumour growth. Earlier reports on the incidence of leiomyoma in various rhinoceros species suggested that tumour development and growth is sex-steroid hormone-dependent [3,4,37,38]. Similar to humans [7,10,11,12,13,14], leiomyoma development in rhinoceros is thought to potentially start upon the onset of ovarian activity at puberty until sex-steroid influence stops at senescence [3]. Our study supports the hypothesis that reproductive tract tumours growth in rhinoceros can be hormone-dependent. Brief monitoring of tumour growth prior GnRF vaccination in one 24-year-old Greater one-horned rhinoceros indicated that tumour volume increased by 46% within just two months. Conversely, cessation of ovarian activity as a result of the GnRF vaccination significantly reduced the diameter of reproductive tract tumours. Moreover, in the absence of sex-steroid hormones, the calculated volume of the tumours decreased by approximately half of the volume prior to the vaccination. In addition, clinical symptoms such as vaginal discharge from necrotic tumour tissue resolved under the treatment. This is the first time GnRF vaccine and subsequent down regulation of reproductive function has been used as symptomatic treatment against reproductive tumour disease in a wildlife species.

GnRF vaccine has been our first choice for down regulating ovarian function in rhinoceroses over other hormone formulations. Alternative considerations would have been long term oral progesterone application or GnRH analogue implants. Oral, synthetic progesterones have been reported before to induce a temporary down regulation of ovarian function in black and white rhinoceros [28,39,40]. However, the objective of oral progesterone use in rhinoceroses has not been a long term down regulation but rather a short-term induction of oestrus [28,39,40]. In humans, progesterone is known to have tumour promoting properties, and uterine leiomyoma show increased expression of progesterone receptors [41]. Therefore, synthetic progesterone was not the first choice as a long term contraceptive in female rhinoceros with a history of extensive reproductive tract tumours. GnRH analogue implants for contraception have been described for exotic felids and giraffe [42,43] and tested in rhinoceroses [Hermes, unpublished data]. The exponential increase in FSH and LH release at the start of this treatment and the technical difficulty of applying the implants into the rhinoceros skin without sedation were considerations favouring the liquid GnRF vaccine.

Breeding management and husbandry of rhinoceroses follows international guidelines [44,45,46]. These guidelines summarize the best possible husbandry tailored to the specific needs of each rhinoceros species. Providing appropriate breeding space, finding matching breeding partners and organizing ‘wedding trips’ poses great challenges in these 1.5–2.5t mammals [44,45,46]. In the past, fertile females or whole breeding programs had to be suspended from reproduction due to a lack of appropriate breeding space [46]. However, such suspension of breeding poses irreversible consequences on the female’s fertility as reproductive pathology may develop [3,4,37,38]. Due to the suspected reversibility of the ovarian down regulation, our results suggest GnRF vaccination as effective contraceptive for younger, still fertile female rhinoceros for a temporary suspension of breeding ≥ 6 month. In mares it was shown that ovarian down regulation was reversible in 92 to 100% of the vaccine treated animals [18,20]. However, here all female rhinoceros treated with GnRF vaccine were of advanced age, the reversibility of the immunocontraceptive and proof of fertility thereafter was not within the scope of this study. Further research is warranted to define the reversibility and safety of GnRF vaccination in female rhinoceroses.

In two Greater-one horned rhinoceros luteal activity measured by faecal 20α-OH-pregnane produced results which did not match with the ovarian morphology as assessed by the ultrasonography. Persistently high 20α-OH-pregnane concentrations were similar to those measured normally during luteal phase or even pregnancy [29,30]. However, ultrasonography of the reproductive organs excluded both possible explanations for elevated 20α-OH-pregnane concentrations. Ovaries of these females showed no luteal structures and both animals were not pregnant. Doubts rose that 20α-OH-pregnane concentrations measured in the faeces did not represent actual, systemic progesterone metabolites, but were rather non-specified substances in the faeces cross reacting with the 20α-OH-pregnane group specific enzyme-immunoassay used.

Measurement of pre-cursor oestrogens and 20-oxo-pregnanes in the serum in two Greater one-horned rhinoceros kept in one institution were in contradiction to 20α-OH-pregnane concentrations measured in the faeces. Precursor oestrogens were baseline and peaked briefly only after 6 months prior to the next vaccine booster. Low serum 20-oxo-pregnane values also suggested ovarian down regulation despite seemingly elevated 20α-OH-pregnane concentrations in the faeces. Based on the ultrasonographic findings, baseline pre-cursor oestrogens and baseline 20-oxo-pregnane values in the serum we concluded that the response to the GnRF vaccine in these two females was similar to the other Greater one-horned and the white rhinoceroses. In addition, we speculated that the erratic and elevated faecal 20α-OH-pregnane concentrations were due to cross reacting, non-specified, phytochemicals in the feed. In fact, analysis of nutritional components fed to these two animals revealed a strong cross reactivity of hay, grass and pellets with the group specific 20α-OH-pregnane immunoassay used in this study. This cross reactivity of the phytochemicals influenced the otherwise reliable detection of progesterone metabolites in Greater one-horned rhinoceros faeces [29].

Phytoestrogens and more recently phytoprogestins were found in a wide variety of foods and plants [47]. Phytoprogestins may induce an agonistic, neutral or even antagonistic effect when directly exposed to progesterone cell receptors [48,49]. Phytoprogestins extracted from red clover were found to bind to intracellular progesterone receptors inducing progestagenic activity [49]. In wild baboons (*Papio hamadryas anubis*) and Phayre’s leaf monkeys (*Trachypithecus phayrei crepusculus*) consuming African black plum (*Vitex doniana*) the containing phytoprogestins induced progestagenic activity acting as natural, temporary contraceptive [50,51]. In chimpanzees (*Pan troglodytes schweinfurthii*) consumption of fruits from the same plant species (*Vitex fischeri*) was associated with an increase of urinary progesterone concentrations far exceeding the normal range of variation. In rhinoceros, phytoestrogens have been shown to bind to oestrogen receptors and are discussed as a potential cause for reproduction failure [52]. In our study we identified plants and pellets which contained substances that cross reacted with our sex-steroid enzyme immunoassays. This resulted in falsely high, erratic 20α-OH-pregnane concentrations while at the same time ovarian function was down regulated and serum 20-oxo-pregnane and faecal oestrogen concentrations were at baseline. This suggested that highly erratic, faecal sex-steroid metabolite concentrations might indicate the presence of non-bioactive, non-steroidal phytochemicals rather than sex-steroid concentrations from the animal’s hormonal activity. However, since only one set of feed samples was analysed in this study and phytochemical concentrations in plants can be variable, levels of cross reactivity may be also subject of change.

In conclusion, GnRF vaccine effectively down regulated reproductive function in female rhinoceros, thus being the first immunocontraceptive protocol described for this species. Immunization against GnRF decreased the size of reproductive tract tumours in female rhinoceros with high tumour load thus representing a symptomatic therapy aimed at the animal’s welfare. Our work is the first to use down regulation of reproductive function as treatment against benign reproductive tumour disease in a wildlife species. Our results further indicate that GnRF vaccine may be a useful contraceptive even for fertile female rhinoceroses, temporarily suspended from breeding, to prevent the formation of reproductive pathology until breeding is resumed. Yet, full reversibility and rhinoceros fertility following GnRF vaccination is unknown and warrants further evaluation.
